# The role of non-invasive devices for the telemonitoring of heart failure patients

**DOI:** 10.1007/s10741-020-09963-7

**Published:** 2020-04-27

**Authors:** A. Faragli, D. Abawi, C. Quinn, M. Cvetkovic, T. Schlabs, E. Tahirovic, H.-D. Düngen, B. Pieske, S. Kelle, F. Edelmann, Alessio Alogna

**Affiliations:** 1 Department of Internal Medicine and Cardiology Campus Virchow-Klinikum, Charité – Universitätsmedizin Berlin, Augustenburgerplatz 1, 13353 Berlin, Germany; 2 Berlin Institute of Health (BIH), Berlin, Germany; 3 DZHK (German Centre for Cardiovascular Research), Partner Site Berlin, Berlin, Germany; 4 Department of Biological Sciences, Rensselaer Polytechnic Institute, 110 Eighth Street, Troy, NY USA; 5 Department of Internal Medicine/Cardiology, Deutsches Herzzentrum Berlin, Augustenburger Platz 1, 13353 Berlin, Germany

**Keywords:** Heart failure, Telemonitoring, Non-invasive, Medical devices

## Abstract

Heart failure (HF) patients represent one of the most prevalent as well as one of the most fragile population encountered in the cardiology and internal medicine departments nowadays. Estimated to account for around 26 million people worldwide, diagnosed patients present a poor prognosis and quality of life with a clinical history accompanied by repeated hospital admissions caused by an exacerbation of their chronic condition. The frequent hospitalizations and the extended hospital stays mean an extremely high economic burden for healthcare institutions. Meanwhile, the number of chronically diseased and elderly patients is continuously rising, and a lack of specialized physicians is evident. To cope with this health emergency, more efficient strategies for patient management, more accurate diagnostic tools, and more efficient preventive plans are needed. In recent years, telemonitoring has been introduced as the potential answer to solve such needs. Different methodologies and devices have been progressively investigated for effective home monitoring of cardiologic patients. Invasive hemodynamic devices, such as *CardioMEMS™*, have been demonstrated to be reducing hospitalizations and mortality, but their use is however restricted to limited cases. The role of external non-invasive devices for remote patient monitoring, instead, is yet to be clarified. In this review, we summarized the most relevant studies and devices that, by utilizing non-invasive telemonitoring, demonstrated whether beneficial effects in the management of HF patients were effective.

## Background

Heart failure (HF) is a clinical syndrome characterized by symptoms such as breathlessness, ankle swelling, and fatigue that may be accompanied by signs such as elevated jugular venous pressure, pulmonary crackles, and peripheral edema [1, 2]. The current definition of HF, as stated by the most recent European Society of Cardiology guidelines, restricts itself to stages at which clinical symptoms are apparent [1, 2]. However, even in the asymptomatic phase, patients may experience already structural or functional cardiac abnormalities (systolic or diastolic left ventricular (LV) dysfunction) that can lead to overt HF [1, 2]. The estimated absolute number of people suffering from HF in the world approaches 26 million, and this widespread pathology can be encountered nowadays both in developed and developing countries [3]. Chronic heart failure (CHF) patients represent one of the leading population at risk of frequent hospitalizations and poor life expectancy, and indeed only 10% of these patients are alive at 10 years from diagnosis [4, 5]. Annually, 1 million patients are hospitalized with a primary diagnosis of HF, accounting for a total Medicare expenditure exceeding $17 billion in the USA every year [6]. Despite dramatic improvements in outcomes with medical therapy, admission rates following hospitalizations remain high, with 20–30% of the patients readmitted after 30 days and > 50% of them readmitted within 6 months after discharge [7, 8]. Because of the high rate of re-hospitalizations, the high mortality, the poor quality of life, and the substantial cost sustained by the national healthcare system, much effort has been made to identify the parameters and risk factors that can help in the prediction and prevention of decompensation events and unnecessary hospitalizations [9, 10]. Several physiological indices of HF severity anticipate serious adverse events such as elevated filling pressures, jugular venous pressure, orthopnea, and echocardiographic filling patterns hospitalizations [9, 10]. Levels of cardiac biomarkers, including natriuretic peptides and cardiac troponins, may also anticipate the readmission risk, particularly if they remain high at hospital discharge [11, 12]. Indicators of neurohormonal activation, including higher levels of circulating catecholamines and renin-angiotensin system metabolites or lower levels of serum sodium, can also identify patients at risk [13, 14]. Increasing diuretic requirements or intolerance of neurohormonal antagonists because of hypotension or renal dysfunction are likely indicators of disease progression and represent an indicator of worsening clinical outcomes [15].

Moreover, the increased burden of atrial or ventricular arrhythmias, the decrease in heart rate variability, and the development of changes in the electrocardiographic traces have been identified as predictors of decompensation events [16–18]. All these parameters, usually investigated in the hospital, are however difficult to obtain when the patients are at home [16–18]. The primary unmet need is indeed the lack of an appropriate and consistent way to predict the decompensation of patients when they are outside the hospital [16–18].

In the recent 10 years, telemedicine, telemonitoring, Mobile Health (mHealth), and Electronic Health (eHealth) have gradually entered the panorama of clinical medicine [19, 20]. The history of telemedicine had started when blood glucose meters, Holter monitoring, event recorders, and 24-h blood pressure monitoring were introduced to the clinical management of patients [19, 20]. It soon became apparent that monitoring patients’ parameters outside the hospital could be a useful way to prevent the occurrence of cardiac decompensation events, especially in a population at risk, such as the one of HF patients [21, 22]. Thanks to the advances in technology and in device miniaturization, a de-medicalization of the data has been achieved [21, 22]. These advances brought a revolutionary change in the final data users, who have eventually become the patients themselves [21, 22]. Nowadays, this is defined as telemonitoring, home monitoring, or remote patient monitoring (RPM). Telemonitoring consists of either a continuous or sporadic monitoring that can be either dependent on the patients’ action or completely independent and automated [21, 22]. The first non-invasive telemedical systems enabled the transfer of physiological data and parameters to the telemedical centers for data integration (e.g., body weight, heart rate, blood pressure, body temperature) by collecting data indirectly via phone calls to the patients [21–23]. Nowadays, more advanced non-invasive systems are able to measure and transfer data measured non-invasively on electrocardiographic (ECG) tracings, oxygen saturation, blood pressure, and physical activity (e.g., pedometer) [24], or invasively, through implantable devices, enabling the transfer of variables such as impedance analysis and pulmonary artery or left atrial pressures [25, 26]. Telemonitoring can also be divided into active and passive telemonitoring. While passive telemonitoring is typical for invasive implantable devices sending either sporadically or continuously data to the receiving physician, active telemonitoring via non-invasive devices involves an action (e.g., a video call) or a self-measurement (e.g., blood pressure measurement) by the patients themselves. The role of implantable telemonitoring devices for multi-parameters [25] or cardiac hemodynamic activity monitoring [27] has been recently established as an effective way to prevent frequent hospitalizations [28]. The role of non-invasive methods for the remote monitoring of HF patients, instead, is still under debate [29, 30]. During the past decade, different studies tried to assess if telemedical interventions and telemonitoring programs would be able to affect mortality and re-hospitalizations of HF patients. Some of the most relevant ones were summarized in Table 1. In this review, we will focus in specific on the role of different non-invasive methods or devices for the remote monitoring of HF patients.

**Table 1:** Overview of the most representative clinical studies analyzing non-invasive remote patient monitoring devices

Author	Year	Type of the study	Number of patients	Endpoints	Method studied	Devices used in study	Results
Cleland—TEN-HMS	2005	Interventional study	426	All-cause mortality or re-hospitalizations for any cause	Structured telephone support or non-invasive home telemonitoring	-Telephone -ECG -Weight scale -Blood pressure monitor	*BENEFICIAL EFFECTS* Lower rate of all-cause mortality
Klersy	2009	Metanalysis	8612	All-cause mortality, hospitalization for any cause or hospitalization for HF	Structured telephone support	-Telephone -ECG	*BENEFICIAL EFFECTS* Lower rate of all-cause mortality, hospitalizations and hospitalizations for HF
Chaudry	2010	Interventional study	1653	All-cause mortality or re-hospitalizations for any cause	Structured telephone support	-Telephone -Weight scale	*NO BENEFICIAL EFFECTS* No differences between the interventional group and the usual care one
Ferrante—DIAL HF	2010	Interventional study	1518	All-cause mortality or hospital admissions 1 year after randomization	Structured telephone support	-Telephone -Weight scale -Blood pressure monitor	*BENEFICIAL EFFECTS* Lower rate of all-cause mortality and hospitalizations for HF
Koehler—TIM-HF	2011	Interventional study	710	All-cause mortality or re-hospitalizations for HF	Non-invasive home telemonitoring	-Mobile phones -ECG -Weight scale -Blood pressure monitor -Pulse/oximeter	*NO BENEFICIAL EFFECTS* No differences between the interventional group and the usual care one
Anand—MUSIC	2012	Interventional study	543	An HF decompensation prediction algorithm with 63% sensitivity, 92% specificity, and a false positive rate of 0.9 per patient-year	Bioimpedance monitoring	-External multi-sensor monitoring system	*BENEFICIAL EFFECTS* An HF decompensation prediction algorithm was developed with sufficient sensitivity and specificity percentages
Angermann—INH study	2012	Interventional study	1007	Time to death or re-hospitalization, HF symptoms and quality of life	Structured telephone support and education	-Telephone -ECG -Weight scale -Blood pressure monitor	*BENEFICIAL EFFECTS* Decreased mortality risk and increased quality of life
Dovancescu—SENTINEL-HF	2015	Interventional study	180	Unplanned HF-related re-hospitalization, HF worsening, major adverse cardiac events, emergency department visits, all-cause re-hospitalization, and death	Bioimpedance monitoring	-A transthoracic bioimpedance vest called FAV (fluid accumulation vest)	*BENEFICIAL EFFECTS* Preventing HF decompensations and reducing hospitalizations
Kotb	2015	Metanalysis	193	All-cause mortality, hospitalization for any cause or hospitalization for HF	Structured telephone support or non-invasive home telemonitoring	-Telephone -ECG & video monitors (applicable to some studies)	*BENEFICIAL EFFECTS* Lower rate of all-cause mortality and hospitalizations for HF, improvement in patients’ quality of life
Ong—BEAT-HF	2016	Interventional study	1437	Readmission from any cause within 180 days after discharge	Structured telephone support and non-invasive home telemonitoring	-Telephone -ECG -Weight scale -Blood pressure monitor	*NO BENEFICIAL EFFECTS* No differences between the interventional group and the usual care one
Inglis	2017	Metanalysis	9332	All-cause mortality or hospital admissions for HF	Structured telephone support or non-invasive home telemonitoring	-Telephone -ECG -Weight scale -Blood pressure monitor	*BENEFICIAL EFFECTS* Lower rate of all-cause mortality and hospitalizations for HF, improvement in patients’ quality of life
Lin	2017	Metanalysis	11,758	All-cause mortality, length of intervention, hospital admission rate, and length of hospital stay	Structured telephone support or non-invasive home telemonitoring	-Telephone -ECG -Weight scale -Blood pressure monitor -Video consultation equipment (applicable to some studies)	*BENEFICIAL EFFECTS* Lower rate of all-cause and HF-related mortality, reduced admission rates, and shortened HF-related length of hospital stay
Koehler—TIM-HF 2	2018	Interventional study	1571	Days lost due to heart failure hospitalization or death	Non-invasive home telemonitoring	-ECG -Weight scale -Blood pressure monitor -Pulse/oximeter	*BENEFICIAL EFFECTS* Lower rate of all-cause mortality and a reduced amount of days lost due to unplanned cardiovascular hospital admissions after a follow-up of 1 year
Nouryan	2018	Interventional study	89	All-cause hospitalization, length of stay, and quality of life	Structured telephone support or non-invasive home telemonitoring	-Telephone -Weight scale -Blood pressure monitor -Pulse/oximeter -Video monitorware	*BENEFICIAL EFFECTS* Lower rate of all-cause hospitalization and length of stay, improvement in patients’ quality of life
Ware	2018	Interventional study	98	Inform the design of telemonitoring services and implementation strategies of similar telemonitoring interventions	Structured mobile phone-based support	-Mobile phone -Weight scale -Blood pressure monitor-*Medly* mobile phone app	*BENEFICIAL EFFECTS* Improvement in patients’ quality of life
Gingele	2019	Interventional study	382	All-cause mortality rate, number and length of HF-related hospital admissions, and number of outpatient clinic visits due to HF during 1 year of follow-up	Non-invasive home telemonitoring	-ECG -Pulse/oximeter-*Health Buddy* electronic device	*NO BENEFICIAL EFFECTS* Tailored telemonitoring did not improve health-related quality of life in HF patients
Zhu	2019	Meta-analysis	10,981	All-cause hospitalization, cardiac hospitalization, all-cause mortality, cardiac mortality, and length of stay	Structured telephone support	-Telephone -ECG -Weight scale -Blood pressure monitor -Pulse/oximeter	*BENEFICIAL EFFECTS* Lower rate all-cause hospitalization, cardiac hospitalization, all-cause mortality, cardiac mortality, and length of stay

The above table covers different studies from the past decade that aimed to assess if telemedical interventions and telemonitoring programs would be able to affect mortality, re-hospitalizations rate, or quality of life of HF patients. The type of study conducted, number of patients involved, endpoints, and devices are included for each study respectively. Finally, the effects of each study were evaluated as to whether they were beneficial or not

### Telephone support

Different ways of remote monitoring HF patients were investigated in the past, and one of the most studied methods involves regular telephone support to monitor symptoms, changes in body weight, and the psychological status of the patients known as telephone support [22, 31, 32]. The DIAL trial, published in 2005, was one of the first randomized trials analyzing the role of telephone intervention against usual care in 1518 outpatients with stable chronic heart failure and optimal drug treatment. This preliminary study was shown to be effective in reducing admissions to hospital for heart failure. In 2010, Ferrante et al. performed an extended follow-up of the DIAL trial and demonstrated that regular phone intervention could improve adherence on diet, weight control, and medications [22]. One year after the intervention, a reduction of 19% for all-cause mortality and all-cause hospitalizations was observed [22]. However, that same year, the group of Chaudry et al. was not able to confirm any beneficial effects of remote telemonitoring (defined as daily calls performed to assess the patient’s health status, symptoms, and changes in body weight measurement) over standard care [31]. The study’s primary endpoint was readmission for any reason or death from any cause within 180 days after enrollment [31]. Secondary endpoints included hospitalizations for HF, number of days in the hospital, and number of all-cause hospitalizations [31]. No ECG data nor other vital parameters were analyzed. Another study, the Interdisciplinary Network for Heart Failure (INH) trial, investigated the role of telephone-based monitoring and education, addressing individual problems of the patients, pursuing networking of healthcare providers, and providing training for caregivers [32]. Even though no reduction in re-hospitalizations was observed, mortality risk and reported patient surrogates of well-being improved significantly, suggesting that individualized care and consideration of non-cardiac problems understandable through telephone support should be integrated into the telemonitoring plans of HF patients [32]. The recent metanalysis by Zhu et al. showed instead that telephone support interventions are likely to reduce the hospitalization for all causes (OR 0.86, 95% CI 0.78–0.96, *P* = 0.006) and the hospitalization due to HF (odds ratio (OR) 0.74, 95% CI 0.65–0.85, *P* < 0.0001), compared with interventions from conventional healthcare [30]. Moreover, it may also impact on cardiac mortality (OR 0.54, 95%CI 0.34–0.86, *P* = 0.009) [30].

### Body weight monitoring

The main reason for the recurrent hospitalizations of CHF patients is a worsening of their disease state, characterized by an excessive decrease of the effective circulatory volume and a consequent mechanism of chronic body water retention [33]. When these patients are admitted to the hospital with body fluids congestion, high doses of diuretics are administered [33]. Further monitoring of the body water balance during the hospitalization is usually achieved via clinical observations regarding the patient’s symptoms (e.g., dyspnea, peripheral edema, and pulmonary crackles), daily assessment of the body weight, and/or documentation of the patient’s fluid input and output [33, 34]. Usually, an improvement of the patient’s symptoms can be achieved in a matter of days; however, in HF patients, ineffective treatment regarding body water retention can lead to clinical instability in terms of pulmonary edema up to death [34]. The paradigm assumed to identify deterioration of heart failure has been defined as an increase of body weight of 2 kg/48 h [34]. Even if this threshold derives more from a consensus agreement rather than from clear clinical evidence, it is still one of the most utilized parameters of remote home monitoring of HF patients [34]. In their clinical trial, Chaudry et al. used weight scales as their primary device to assess data. Using this evaluation of patients’ health status, the study was not able to confirm any beneficial effects from telemonitoring [31]. This study did not include any ECG analysis nor other vital signs, supporting the fact that monitoring patients’ body weight alone may be less beneficial than utilizing a combination of different parameters [31]. Another study that was not able to prove any significant decrease in re-hospitalizations nor mortality in the intervention group was the BEAT-HF [35]. Signals of daily changes in weight did not prove adequate warning of impending decompensation [35]. The presence of multiple parameters and multiple devices, instead, seems be more beneficial in detecting better decompensation events. An example of a study in which body weight monitoring combined with additional parameters from multiple devices was conducted in 2018 by Nouryan et al. [36]. Devices used in this study included a weight scale, a blood pressure monitor, pulse/ oximeter, and a video monitor [36]. This study confirmed beneficial effects from telemonitoring including lower rate of all-cause hospitalizations and length of stay, as well as improvement in patients’ overall quality of life [36].

### ECG monitoring

Among the different non-invasive technologies, ECG monitoring remains a relatively under-investigated topic for the remote monitoring of HF patients [29]. Electrocardiographic technology has become more and more prevalent since its development in the 1950s [20]. ECG examinations have moved from in-hospital to ambulatory settings and nowadays have the possibility of becoming more mobile and accessible [20]. With the emergence of the Internet, Wi-Fi, cellular networks, and broad-band transmission, it is easier to perform such examinations remotely today [20]. Even if recently the potential for ECG examinations being used digitally in combination with smartphone applications and miniaturized devices or wearables is staggering [20, 37, 38], there is more than ever the need of further clinical randomized trials for such new devices [39]. In a study by Cleland et al., ECG data transmission was significantly associated with reducing hospitalizations due to HF when compared with usual care [40]. In a study by Villani et al. which analyzed HF patients at high risk of relapse, the regular acquisition of simple clinical information as well as the possibility for the patients to contact the clinical staff and having access to the ECG data, produced better psychological status, quality of life, and reduced hospitalizations for HF patients [41]. A metanalysis by Kotb in 2015 focused primarily on assessing ECG tracings, telephone support, and video monitors to abstract data for the study [29]. The prominent role of the ECG in this analysis proved beneficial as the study showed substantial beneficial positive effects including lower rates of all-cause mortality and hospitalizations, and an improvement in quality of life for HF patients [29]. The extent to which ECG is useful and to which type of HF patients should be applied is, however, still to be completely determined. For instance, in a study conducted by Ferrante et al., the intervention group did not use the ECG as a parameter, yet the effects of the study were still shown to be beneficial in terms of lower rates of all-cause mortality and hospitalization in HF patients [22]. Similar to the study by Ferrante et al., the study conducted by Nouryan et al. in 2018 had success by using different telemonitoring methods apart from ECG data [36]. Despite not having ECG measurements, this version of telemonitoring showed beneficial effects including lower rates of all-cause hospitalization, length of stay, and improvement in patients’ quality of life [36]. Much literature has been published on the utility of the ECG for predicting HF worsening in chronic HF patients. The parameters which were found to predict the risk of a heart failure decompensation event are summarized in Table 2.

**Table 2:** Overview and definition of the most studied ECG parameters predicting decompensation events in cardiological patients

Parameters	Definition
Reduced heart rate variability [18]	Reduction in time domain differences between day and night observed at Holter monitoring
Increased heart rate [42]	Tachycardia and increase in the basal heart rate
QRS/T angle increase [43]	Increase in the repolarization axis angle (> 60° for women and > 120° for men)
Atrial and ventricular tachyarrhythmias [19, 44]	Atrial flutter and fibrillation and ventricular non-sustained and sustained ventricular tachycardia
Increased ECG LV mass [20, 45]	More than 70 ± 9 g/m^2^ in men and 61 ± 8 g/m^2^ in women
QT prolongation [46]	QTc > 450 ms (males) QTc > 460 ms (females)
Increased QRS duration [47, 48]	QRS > 100 ms with or without complete and/or incomplete bundle branch blocks (left or right)
LV strain [17, 49, 50]	ST segment depression and T wave inversions
An old silent myocardial infarction [51]	Novacode Criteria published in the article by Rautaharju et al. [51]

ECG data and parameters allow physicians to have a better psychological status of the patient for better predictions. The preceding table displays the parameters which were found to predict the risk of a heart failure decompensation. Each individual risk is accompanied by a definition for reference

### Bioimpedance monitoring

Body weight assessment is an imprecise estimation of the body fluid composition since it can be influenced by a wide variety of factors [52]. Bioimpedance analysis is a way to measure the body composition, by assessing the lean and fat body mass, total body water, and extracellular/intracellular water [52]. While a single measurement of the patient’s fluid status is not particularly useful, a relative change of the patient’s fluid balance is crucial in HF patients [52]. For this reason, a sensible way of analyzing the patients’ body water overload was firstly obtained invasively through a current generated from the pacing wires of pacemakers and defibrillators [53, 54]. This kind of measurement estimates the fluid overload locally inside the thorax of the patient. As an example, Medtronic developed a CRT-ICD device which is equipped with an internal impedance-meter called Opti-Vol. This feature for bioimpedance analysis has been evaluated in the DOT-HF trial with the objective to test whether Opti-Vol would affect the clinical outcomes of CHF patients [55]. Interestingly, the intervention arm has been shown to be associated with a borderline statistically significant increase in the primary endpoint composed by all-cause mortality and HF hospitalizations compared with the standard care one. This increase was mostly due to increased HF-related admissions. This can be explained by the fact that an increase in the incoming additional diagnostic data obtained from the Opti-Vol method provided an increase in HF hospitalizations [55]. To implement a possible way of monitoring the fluid status of HF patients with external devices instead, some studies were conducted to assess the possible utilization of non-invasive bioimpedance analysis for the remote management of HF patients [52]. One interventional study, called MUSIC (Multi-sensor Monitoring in Congestive Heart Failure), was initiated to develop and validate an algorithm for prediction of impending acute heart failure decompensation with the use of different physiological parameters, including bioimpedance analysis, obtained from an external device adhered to the chest [56]. Five hundred forty-three HF patients with an ejection fraction less than 40% and a recent HF admission were recruited. They were remotely monitored with a multi-sensor device for 90 days [56]. A total of 314 patients were included in the analysis: 114 in the development cohort, and 200 in the validation cohort [56]. A multi-parameter HF detection algorithm was developed from the obtained data in the development cohort. This algorithm had a 65% sensitivity and 90% specificity for the detection of HF events in that cohort and met the pre-specified endpoints in the validation cohort with a sensitivity of 63% and specificity of 92% [56]. However, whether this method would affect the clinical outcome of CHF patients was not studied and is still yet to be determined [56]. A recent study, called SENTINEL-HF, examined a transthoracic bioimpedance vest called FAV (Fluid Accumulation Vest) in 180 patients hospitalized for HF [57]. The patients were trained to autonomously perform daily bioimpedance measurements and transmit them via their smartphone to the clinic of reference [57]. This preliminary study identified that the use of FAV allowed to predict the occurrence of hospitalizations up to 7 days in advance in the intervention group [57]. However, further studies are needed to assess the role of bioimpedance analysis in preventing hospital admissions.

### Applications, software, and new technologies

Digital health is an increasingly emerging medical field [58]. Advances in technology were translated into healthcare due to access of an immense amount of data, to new software platforms, and even to the use of Artificial Intelligence (AI) [59]. The development of digital health has been recognized by the European Society of Cardiology with the recent establishment of the *Digital Health Virtual Journal* in August of 2019 [58, 59]. This journal was established to give information about the digital revolution in cardiology. This momentum of use of digital health in clinical practices and research is expected to rise in the years to come [58, 59]. Already many examples of digital health have been seen in several studies through their use of technology in relation to HF patients as well as devices used in telemonitoring methods [58, 59]. An example is given by the pilot study investigating a device called *MedSentry*, a remotely monitored electronic pillbox, that alerts patients when it is time to take their medication and connects patients with caregivers in case the medication was not taken, showed a reduction in all-cause hospitalizations and all-cause length of stay in the intervention group [60]. Ware et al. conducted another study which took a different approach to telemonitoring by using the *Medly* mobile phone application that allows patients to record their blood pressure, body weight, and symptoms on a daily basis [61]. Patients received an automated phone call if they had not taken their readings before 10 AM to encourage participation [61]. Assessing the user inputs, *Medly*’s algorithms can output self-care messages as well as clinician alerts based on patient-driven target ranges [61]. The study’s aim was to integrate *Medly* as part of the standard of care of HF patients. Gingele et al., instead, made use of an electronic device called *Health Buddy* [62]. This device, made up of a display screen and four buttons, presents the patient with health education and self-care support and additionally relays questions about the patient’s symptoms that are then collected by a protected server [62]. These studies are an example of how applications, software, and other devices represent the new frontiers of telemedicine to come.

More and more devices, often referred to as wearables and being a part of mobile health (mHealth), are taking place in our daily life. These biosensing products come in a variety of different forms and are often integrated into clothing or accessories (e.g., watches). Wearables offer the possibility of continuously collecting functional or physiological data outside the hospital [39]. Previous evaluations of wearables in patients with heart failure mainly focused on pedometers and activity trackers showing for example that wireless mobility monitoring after cardiac surgery was feasible and practical [63], or that cardiac tele-rehabilitation via a call center can support walking activity using pedometers leading to an incorporation of step count into the intervention [64]. In consequence, wearables not only have the capacity of assessing data but also become components of clinical trials in the form of interventions and endpoints [39]. Furthermore, Chan et al. screened heart failure patients for atrial fibrillation by using handheld ECG recording devices (*AliveCor* device, San Francisco, CA) that transmits to a smartphone application, showing the feasibility and identification of a significant proportion of patients with newly diagnosed atrial fibrillation [65]. Further technologies as for example vests measuring thoracic fluid content [66] and ballistocardiogram or seismocardiogram devices measuring whole-body movements and chest wall vibrations are emerging in order to detect cardiac decompensation events [67]. Overall, wearables have the possibility to improve care and outcomes in heart failure patients; however, the current data is limited to feasibility studies and small randomized controlled trials [39].

#### Lung ultrasound

Lung ultrasound (LUS) became a valuable diagnostic tool in patients with heart failure over the last decade. As a point of care test, it emerged as a simple and non-invasive tool for the detection of pulmonary congestion and cardiac filling pressures [68, 69]. Studies have shown a fast learning curve and high inter-observer agreement [70, 71]. European guidelines support its use in patients with acute heart failure [1, 72]. Furthermore, LUS is even more effective to detect pulmonary congestion than clinical examination, chest X-ray or NT-proBNP measurements [73, 74]. The technique is based on the detection of B-Lines which is defined as a kind of comet-tail artifact indicating subpleural interstitial edema. The number of B-lines correlates with the presence of extravascular lung water and identifies patients with worse outcome very well [69, 70]. This has been shown in both in- and outpatient heart failure cohorts with persistent B-lines at discharge [75–77]. As a first randomized control trial, the LUS-HF study evaluated the use of lung ultrasound to guide the ambulatory follow-up over a period of 6 months in 123 patients which were hospitalized due to heart failure [78]. Patients were randomized either to the LUS-guided group or the standard care group. In both groups, lung ultrasound was performed with a pocket ultrasound device (*VScan*; GE Healthcare, Chicago, IL, USA) [78]. Only patients were blinded to their group assignment. The treating physicians were not blinded; however, the LUS results were withheld in the standard care group. Diuretic therapy was modified if number of B-lines recorded by LUS surpassed more than 3 B-Lines in 8 chest zones. The study could show that patients in the LUS group had a significantly improved combined primary endpoint of urgent visits, hospitalization for worsening heart failure, and all-cause death over a 6-month follow-up [78]. In addition, there was no difference regarding the risk of adverse events between both groups. As a non-invasive, easy to handle, and cost-effective method, LUS might be a broadly applicable approach to monitor cardiac decompensation in heart failure patients. Of course, further and larger trials are needed to prove that LUS-guided therapy management of heart failure patients in different settings is truly beneficial and to better define B-line cutoff values, establish treatment strategies, and identify subgroups that are more likely to benefit [79, 80]. A systematic review by Swamy et al. could show that HF nurses as well as other healthcare providers can quickly optimize the performance of LUS and interpret B-Lines and pleural effusions as signs of pulmonary congestion in patients with heart failure [79]. HF nurses are well-established and important players in the management and care of HF patients. The incorporation and performance of LUS by trained nurses may further improve care and reduce costs in this cohort of patients. Several studies support the possibility that nurse-performed ultrasound examinations is highly valuable to stratify ambulatory HF patients according to risk as well as improve their management likewise applicable in regions of limited resources [76, 81–83].

#### Natriuretic peptides

Natriuretic peptides, as the B-type natriuretic peptide (BNP) and amino-terminal pro-B-type natriuretic peptide (NT-proBNP), are biomarkers which are used for the diagnosis of heart failure. They reflect severity of heart failure and are also significantly associated with adverse outcomes as well as prognosis of patients with heart failure [84, 85]. If serial BNP measurements are useful to guide heart failure therapies is still uncertain. Several clinical trials of different size and design regarding this question have shown mixed [84–87]. In a promising meta-analysis by Troughton et al. natriuretic peptide-guided therapy of patients with heart failure, a reduction of all-cause mortality in patients aged < 75 years and overall reduction of heart failure and cardiovascular hospitalization were shown [88]. However, the succeeding GUIDE-IT trial found that NT-proBNP–guided therapy was not more effective than standard care in improving outcomes in patients with HFrEF [89]. The trial was stopped for futility due to no significant differences between the NT-proBNP guided group and the usual care group regarding primary and secondary endpoints. The primary end point consisted of a composite of time-to-first heart failure hospitalization or cardiovascular mortality. The PRIMAII trial (Can NT-ProBNP-Guided Therapy During Hospital Admission for Acute Decompensated Heart Failure Reduce Mortality and Readmissions?) demonstrated similar results showing that a NT-proBNP guided heart failure therapy with a reduction of NT-proBNP < 30% did not improve 6-month outcomes in conditions of acute heart failure [90]. The Heart Failure Outpatient Monitoring Evaluation (HOME) study on heart failure patients with reduced ejection fraction and recent heart failure hospitalization suggested that daily home BNP measurements could predict impending clinical deterioration [91]. Earlier trials used infrequent monitoring of natriuretic peptides, potentially underestimating its actual benefit. The HOME study was designed as a randomized clinical trial to discourse whether daily BNP measurement integrated into a home monitoring system improved outcome compared to a home monitoring system without daily BNP measurements and a third arm consisting of a usual care approach [91]. The study was terminated prematurely because of a low enrolment rate, low event rate regarding the primary endpoint, and the lack of a formal algorithm to interpret and act upon changes in BNP trends [91]. As a consequence, the data from all study arms was pooled and an analysis as a single observational study was performed. The HOME study could confirm that BNP home measurements are safe and feasible which is consistent to the previous published HABIT (Heart Failure Assessment With BNP in the Home) trial [91, 92]. Regarding the used remote home monitoring device, BNP measurements were realized by the *Alere™ Heart Check* system with a finger-stick self-testing sample at home. At the time of weight measurement, subjects were instructed to perform their daily BNP analysis each morning before breakfast and morning medication. The weight of the patients was assessed by digital scales that transferred information wirelessly to the *HeartCheck* system. Furthermore, subjects had to answer five questions concerning typical heart failure symptoms for daily clinical status evaluation BNP measurements showed a high day-today variability which increased with a prolongation of measurement intervals [42, 93]. McDonald et al. calculated a moving average filter (fBNP) to reduce day-to-day variations and assess weekly changes, showing that fBNP was able to predict an emerging acute cardiac decompensation [93]. Interestingly, secondary results of the HOME trial showed differences between patients with HFpEF or HFrEF regarding their BNP and weight values before decompensation [43]. Randomized clinical trials are needed to determine if changes in daily BNP measurement have an impact on outcome. We summarized the advantages and disadvantages of the emerging and further new applications in Table 3.

**Table 3:** Advantages and disadvantages of emerging wearables and new technologies for remote patient monitoring utilization

Author	Device	Method	Study design	Patientsstudied	Primary endpoint	Results	Pros	Cons
Amir et al. 2017	ReDS™Wearable System	Measurement of dielectric tissue properties through low power electromagnetic signals across the chest to estimate lung fluid volume by a wearable vest.	Observational	50	Readmission due to HF	Reduction of readmission of 87% after 3 months	Portable Easy to use Fast assessment (90s) Can be worn on top of clothing Minimal patient collaboration	External device relatively big
Inan et al. 2018	Seismocardiogram sensing patch	Seismocardiography (measurement of chest wall vibrations)	Feasibility	45	6-min walk test	Seismocardiography by a wearable patch can assess compensated and decompensated HF states	Small, lightweight wearable patch	May be inconvenient due to a daily required performance of a 6 min walk
Elian et al. 2016	Shoe-mounted pressure sensors	Shoe-integrated sensors for body weight estimation	Feasibility	161	Accuracy of weight measurement	Weight measurement from shoe-integrated pressure sensors are inaccurate	Easy to use Comfortable for the patient	Imprecise
Rivas-Lasarte et al. 2019	VScan®; GE HealthcarePortable Ultrasound	Lung ultrasound	Randomized clinical trial	123	Composite of urgent visits, hospitalization for worsening HF and death from any cause	Tailored LUS-guided diuretic treatment of pulmonary congestion in this proof-of-concept study reduced the number of decompensations and improved walking capacity in patients with HF.	Established method Cost-effective Fast learning curve Portable Applicable by non-experts	No self-measurement So far not integrated into home monitoring systems
McDonald et al. 2018	Alere™ HeartCheck	Blood sampling of BNP through a finger-stick device	Randomized clinical trial	107	The primary endpoint was a composite of HF-related death, hospital admission due to acute decompensated heart failure.	The feasibility of home BNP measurement is demonstrated and the potential value of fBNP is shown as an index of emerging clinical deterioration.	BNP is a sensitive parameter for detecting heart failure decompensation The device is minimally invasive and easy to use	Assessment of the clinical value of the outcome is still required. BNP measurement is not sufficient, and patients need to additionally weigh themselves everyday

## Discussion

### Is non-invasive telemonitoring beneficial or not?

Despite all the new advances in therapy, the management of CHF patients remains a massive burden for the healthcare system [44]. This is only worsened by the increasing lack of medical doctors with expertise in HF management, becoming a relevant issue particularly in rural areas of Countries such as Germany or the UK [44]. Therefore, telemedical care has been recently proposed as a potentially efficient and cost-effective way to provide care and improve the outcome of HF patients [44]. Many studies have been conducted, providing both invasive and non-invasive solutions for HF patients [44]. In recent years, a broad consensus concerning the favorable prognostic impact of implantable hemodynamic monitoring has been reached. Invasive telemonitoring, such as via pulmonary artery monitoring (*cardioMEMS™*) [45] or via ICD multi-parameter monitoring (the IN-TIME approach) [25], has therefore already been integrated in the 2016 ESC HF guidelines with a class IIb level of recommendation [1]. A certain disagreement, instead, is still present about the effectiveness of non-invasive methods in reducing patients’ hospitalizations, and this type of monitoring is not part of any guidelines or consensus agreement yet [46, 47].

In the last 10 years, different randomized clinical trials were performed to finally reply to this question and to prove the utility of non-invasive RPM [23, 24, 35]. From 2008 until 2011, a large randomized multicenter trial, the Telemedical Interventional Monitoring in Heart Failure (TIM-HF), was designed to investigate whether RPM would reduce mortality and hospitalizations in ambulatory chronic HF patients compared to usual care [23]. External devices for ECG, blood pressure, and body weight measurements were connected via Bluetooth to the patient’s home, and information were sent to the Center Monitors continuously 24/7 [23]. The primary endpoint was death from any cause. The secondary endpoint was a composite of cardiovascular death and hospitalization for HF [23]. The results of TIM-HF suggest that when RPM is applied to stable, optimally treated, ambulatory chronic HF patients, a reduction in mortality and re-hospitalizations is not evident. However, this study confirmed that non-invasive telemonitoring improves the quality of life of HF patients [23]. Another large trial, the BEAT-HF that enrolled 1437 participants investigated the role of combined health coaching telephone calls and telemonitoring [35]. The primary outcome, namely readmission for any cause within 180 days after discharge, was not different between the intervention group compared to the standard care group [35]. In a secondary analysis, there were no differences in 30-day readmission or 180-day mortality, but there was a significant difference in the 180-day quality of life between the intervention and usual care groups [35].

Published in 2018, the Telemedical Interventional Management in Heart Failure II study (TIM-HF II), the more extensive follow-on study to the TIM-HF trial, by Koehler et al., was the first non-invasive randomized-controlled trial which showed an improvement in all-cause mortality by RPM in patients with heart failure [24]. The study was conducted from 2013 till 2017 and 1571 patients with heart failure were included and randomly assigned either to the remote patient monitoring group (*n* = 796) or to the standard care group (*n* = 775) [24]. The non-invasive telemonitoring system, which was installed in the patient’s home, consisted of a three-channel ECG, a blood pressure monitoring device, a pulse oximeter, and a weight scale [24]. Patients were equipped with a mobile phone to contact the telemedical center in case of emergencies [24]. Likewise, a monthly follow-up was achieved via structured phone interviews with the patients [24]. The telemedical center provided patients with 24 h a day and 7 days a week of medical support led by physicians and heart failure nurses, being able to act immediately according to the patient’s specific risk profile [24]. The interventions initiated by the telemedical center involved changes in medication, initiation of ambulatory assessments, hospital admission, and educational activities [24]. The primary endpoint of days lost due to unplanned cardiovascular hospital admissions and all-cause mortality (in %) showed a significant advantage after a follow-up of 1 year for patients in the RPM group compared to the control group (hazard ratio 0.80; 95% CI 0.65–1.00; *P* = 0.046) [24]. Per year, patients in the RPM group lost a mean of 17.8 days (95% CI 16.6–19.1) compared with 24.2 days (95% CI 22.6–26) per year for patients assigned to the control group [24]. In addition, the all-cause death rate per 100 person-years of follow-up was significantly reduced in the RPM group [7.86 (95% CI: 6.14–10.10)] compared to the control group [11.34 (95% CI: 9.21–13.95)] per 100 person-years of follow-up (hazard ratio 0.70; 95% CI 0.50–0.96; *P* = 0.0280) [24]. There was no significant difference in cardiovascular mortality between the two groups (hazard ratio 0.671, 95% CI 0.45–1.01; *P* = 0.056) [24]. These results show that non-invasive telemonitoring can lead to an improvement in prognosis and a reduction of hospital admissions in high-risk patients [24].

Besides clinical trials, metanalyses on telemonitoring and telephone support have been performed, suggesting that RPM could provide better clinical outcomes than usual care, with a reduction in mortality and hospital admissions observed [48–50]. In the Cochrane Review by Inglis et al. in 2010, 25 full peer-reviewed studies on non-invasive telemonitoring were included in the analysis and incorporated 8323 patients [48]. In addition to examining the impact on heart failure-related hospitalization and mortality, the review also considered the quality of life (QOL), acceptability of the systems, and cost efficacy [48]. Telemonitoring reduced all-cause mortality (relative risk (RR) 0.66, 95% confidence interval (CI) 0.54–0.81, *P* < 0.001), whereas telephone support demonstrated a non-significant reduction (RR 0.88, 95% CI 0.76–1.01, *P* = 0.08) [48]. Both telemonitoring and telephone support produced significant reductions in heart failure-related hospitalizations (RR 0.79, 95% CI 0.67–0.94, *P* = 0.008 and RR 0.77, 95% CI 0.68–0.87, *P* < 0.001, respectively) [48]. In a further investigation of the beneficial qualities of telemonitoring, Inglis et al. performed a more recent study in 2017 which included the latest clinical trials and showed that the telemonitoring interventions improved quality of life, reduced the costs, and were acceptable for the patients [50]. Telemonitoring showed a reduction of 20% of all-cause mortality compared to standard of care [50]. For Inglis et al., improvements in drug prescriptions, patient-knowledge, and self-care and functional classes were observed [50]. In a further metanalysis conducted by Lin et al. in 2017, devices studied included ECG, weight scale, blood pressure monitor, and video consultation equipment [51]. The number of participants consisted of a total of 11,758 patients, of which 5935 subjects belonged to the telemedicine groups, while 5823 subjects were in the control group [51]. The outcomes of the study supported telemonitoring being beneficial to patient’s health, specifically resulting in a lower rate of all-cause and HF-related mortality, reduced admission rates, and shortened HF-related length of hospital stay for the telemedical group [51]. The most recent metanalysis published by Zhu et al. and incorporating 10,981 patients displayed the beneficial effects of telemonitoring such as lower rates of all-cause hospitalization, cardiac hospitalization, all-cause mortality, cardiac mortality, and length of hospital stay, adding an important contribution to the discussion [30].

Telemonitoring as an intervention has been shown to reduce symptoms and improve quality of care through frequent monitoring of patients at home [24, 28]. This, however, needs to be coherent with an easy utilization of these devices that need to be portable and usable for everyone. Of extreme relevance is the consideration that most of the population with CHF are patients older than 65 years [94]. The metanalysis by Inglis tackled this subject and revealed that no differences in age ranges affected the outcome and the adherence to telemonitoring in the studies analyzed [94]. Ware et al. conducted an additional study measuring patient adherence to a mobile phone-based HF telemonitoring program in 2019 [95]. The results of this study showed the highest and most consistent adherence among elderly patients, while the adherence of younger patients decreased over time [95]. These studies confirm that elderly patients are able to adapt to technology for healthcare purposes [94, 95].

The more frequent assessment of the patient status and an earlier recognition of decompensation events through RPM represents a recognized benefit [96]. Telemonitoring provides the patients with a structured disease management process and can be self-empowering, meaning that the patient is actively involved in controlling his health status and lifestyle [96]. This is consequent and in line with the role of the patient that has entirely changed in recent years, switching from a passive to an active role given by the spread of medical knowledge through the internet and a growing attention to personal health [96]. Eventually, a further positive aspect of telemedical solutions is represented by the incorporation of human interaction, such as between the patient and the physician, or the nurse, for example via telephone, where this contact can also detect depression, which is a known risk factor of poor outcome in HF patients [96].

### Economic aspect and reimbursements

Whether telemonitoring is a tool to decrease the costs of care for HF patients mainly by reducing hospitalizations and lengths of hospital stay has been a matter of discussion in different papers [97–101]. In a randomized controlled study by Dendale et al., the total hospitalization cost for heart failure and/or renal failure in a group of HF patients was almost double in the usual care group (1458 + 3420 €/patient) as compared with the telemonitoring group (902 + 2277 €/patient), even if this difference was not found significant (*P* = 0.23) [97]. In a systematic review performed by Seto, a cost comparison analysis was performed between telemonitoring and usual care in HF patients [99]. The studies included in the review showed costs reduction ranging from 1.6 to 68.3%, mainly attributed to a reduction in heart failure hospitalizations [99]. Cost reductions were mainly attributed to reduced hospitalization expenditures. Only one study discussed the impact of HF telemonitoring on direct patient costs. The study found a 3.5% lower travel cost for patients using telemonitoring compared to those in the usual care group. The single study that was found for indirect costs described the willingness to pay for telemedicine by patients with HF (55% of the patients with HF were willing to pay $20 to access telemedicine, and 19% were willing to pay $40) [99].

The group of Blum et al. has instead obtained contrasting data, and they mostly concentrated in understanding the effects of home monitoring on medical costs, 30-day re-hospitalization, mortality, and health-related quality of life [100]. Telemonitoring did not result in lower total costs and a decrease in 30-day readmission rates for the first year and did not result in decreased total costs or better outcomes [100]. A metanalysis by Klersy et al. demonstrated that RPM compared to usual care generates a cost-saving combined with a quality-adjusted life years (QALYs) gain of 0.06 suggesting that RPM is a “dominant” technology over existing standard care [101]. In the budget impact analysis, the adoption of an RPM strategy entailed a progressive and linear increase in costs saved [101]. The difference in costs between RPM and usual care ranged from 300 € to 1000 € per patient per year based on the Diagnosis Related Group (DRG), favoring RPM [101]. The higher the DRG, the greater the saving [101].

### What is the evidence on target patient population and optimal measure frequency?

The optimal timing of telemonitoring is not yet established. A meta-analysis by Nakamura et al. suggests that a high measurement frequency (> twice a week) is more effective in reducing mortality than a lower measurement frequency (≤ once a week) [102]. A relevant argument in favor of a daily monitoring if vital signs is related to the potential risk of sudden heart failure of these patients [102]. Moreover, the strongest benefit can be achieved if daily telemonitoring is linked to a specific action, such as medication adjustment or increased medication adherence. Of importance, a medical doctor or a specialized nurse should always be present to review the parameters telemetrically in order to prescribe and adjust any type of medication. Even in the presence of advancements in artificial intelligence and automated software, the role of a specialized figure is still unavoidable. In particular, specialized HF nurses are known to play a crucial role for the management of HF patients, specifically in cases of cardiac rehabilitation programs or home-based telemonitoring [103]. The turning point is driven by the fundamental influence that the specialized nurses can provide in improving quality and delivery of care to the HF patient [104], going beyond the supervision of vital parameters by positively affecting the patients’ self-care and psychological status [105].

Which type of patients should benefit more from non-invasive telemonitoring and at which timing this should be applied, represent still open themes for discussion. The White Paper from the Heart Failure Society of America Scientific Statements Committee states that external devices should be focused on populations at higher risk [47]. However, the exact criteria for selection of patients at risk and in need of telemonitoring are still far from being widely approved. The most relevant criteria refer to symptomatic patients (NYHA class 3), with recent hospitalizations, a history of body fluids overload and a lack of appropriate medication adherence [106, 107]. Patients with very advanced disease or with significant renal insufficiency may be too ill to achieve benefit from RPM. For this reason, it is reasonable to think that telemonitoring should be provided as a solution for patients at high risk and mostly right at the hospital discharge, since there is evidence that the first 30 days are characterized by high morbidity and mortality for HF patients. This should be however personalized by considering patients characteristics and related comorbidities, while justifying the extension of a telemonitoring plan up to 6 months in individual cases (such as patients prone to fluid overload or lacking a rigorous medication adherence).

### What needs to be changed or improved?

An answer that still needs to be fully replied is to which type of HF patients shall be telemonitoring applied and which type of non-invasive devices shall be utilized. First of all, the accuracy and the clinical value of the specific medical devices should be considered. An example can be given by the utilization of standard weight scales [98]. Even if the remote monitoring of the patient’s body weight is currently considered not sensitive enough to detect early cardiac decompensation events, it is still one of the most used methods for controlling the patient’s status remotely, mainly because of the affordability and of the vast spread of standard weight scales [98]. It should be, instead, substituted by more accurate techniques for the measurement of the patients’ volume status [98]. One possible solution for a new sensory technique may be through the use of bioimpedance analysis. While weight scales are not sensitive enough to detect early cardiac decompensation events, bioimpedance analysis is able to provide a non-invasive approach in predicting volume overload before a given volume overload affects body weight [98]. Although being more accurate than weight scales, there are still limitations to bioimpedance analysis. One of them is related to the water distribution in the body that changes with the patient’s degree of obesity and on populations-specific bioimpedance equations [98, 108]. Morbidly obese patients experience a relatively high amount of extracellular water and total body water. This inconstant hydration factor can lead to underestimation of the percentage of body fat and an overestimation of fat-free mass [98].

Currently, the profile of patients who can potentially benefit from telemedicine should be further investigated in adequately powered randomized clinical trials [46]. In HF patients, the presence of comorbidities, such as chronic obstructive pulmonary disease, chronic kidney disease, or anemia, for example, can negatively affect their outcome [98]. The assessment and the measurement of these comorbidities will need new sensory techniques and new specific devices that have to be addressed to the specific patients’ characteristics [98]. Since nowadays the spread of portable and affordable external devices is increasing, many of which connected to smartphones, more clinical trials on the usefulness of these devices would be helpful to assess their actual clinical utility [46]. Much literature has been published about the role of ECG for predicting cardiac decompensations in chronic HF patients (Table 2). However, these parameters are rarely utilized to predict the patients’ decompensation events remotely [46]. For this reason, either new devices or new algorithms are needed to improve the diagnosis and the risk stratification workflow of HF patients remotely [46].

**Fig. 1:**
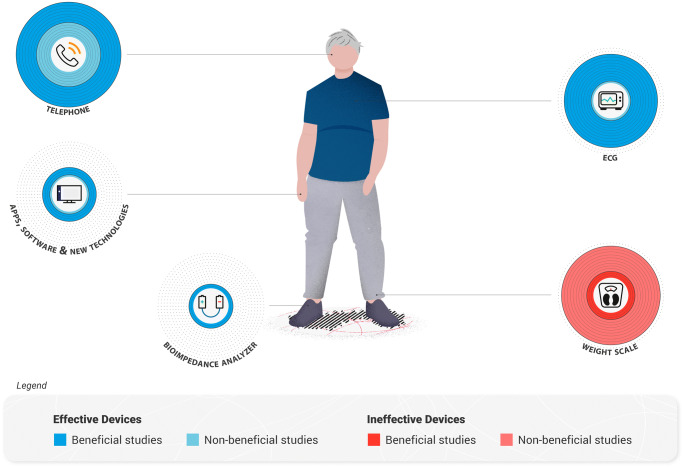
Overview of non-invasive devices commonly used for the remote monitoring of heart failure patients. The devices are sized according to how much have been investigated in the literature, with the larger circles corresponding to the most studied devices. The devices are also categorized by their effectiveness in evaluating a patient’s health status: effective (blue) or ineffective (red)

In conclusion, several study techniques and their applicable devices have been discussed and evaluated for their usefulness in monitoring remotely HF patients and have been summarized in Fig. 1. Telephone support is a useful technique that allows patients to have contact with a healthcare professional and allow frequent updates, yet this kind of measurement alone is not specific enough to make predictions about the patients’ health. Weight scales used to monitor body weight changes are not found to be useful as the patient’s weight is currently considered not sensitive enough to detect early cardiac decompensations. ECG monitoring is an extremely useful technique that provides important patient data yet remains still under-investigated, mainly regarding which population of HF patients would benefit the most. Bioimpedance analysis is a promising form of telemonitoring with great potential; however, there is a lack of evidence for such a technology for remote patient monitoring. Moreover, until now, bioimpedance analysis has been contraindicated in patients implanted with pacemakers, even if numerous studies were able to demonstrate a high level of safety and no adverse events in this population of patients [109–111].

Summing up, the use of only one parameter or device is definitively insufficient [112], while the combination of several parameters and different targeted devices represents the ideal approach, as in the TIM-HF II trial [24].

## Conclusions

Modern advances in technologies have created new opportunities to provide telemedical care as an adjunct to medical management of patients with HF. Non-invasive telemonitoring can reduce morbidity and mortality in these patients as demonstrated by different meta-analyses and the recent clinical trial TIM-HF 2. The debate on the utility of non-invasive devices for home telemonitoring should concentrate on obtaining parameters and prediction algorithms based on more personalized risk profiles of HF patients.
